# MiR-1248: a new prognostic biomarker able to identify supratentorial hemispheric pediatric low-grade gliomas patients associated with progression

**DOI:** 10.1186/s40364-022-00389-x

**Published:** 2022-06-17

**Authors:** Giuseppina Catanzaro, Zein Mersini Besharat, Andrea Carai, Natalie Jäger, Elena Splendiani, Carole Colin, Agnese Po, Martina Chiacchiarini, Anna Citarella, Francesca Gianno, Antonella Cacchione, Evelina Miele, Francesca Diomedi Camassei, Marco Gessi, Luca Massimi, Franco Locatelli, David T. W. Jones, Dominique Figarella-Branger, Stefan M. Pfister, Angela Mastronuzzi, Felice Giangaspero, Elisabetta Ferretti

**Affiliations:** 1 Department of Experimental Medicine, Sapienza University of Rome, Viale Regina Elena 324, 00161 Rome, Italy; 2 Department of Neurosciences, Neurosurgery Unit, IRCCS Bambino Gesù Children’s Hospital, Rome, Italy; 3 Division of Pediatric Neurooncology, Hopp Children’s Cancer Center Heidelberg (KiTZ), German Cancer Consortium (DKTK) and German Cancer Research Center (DKFZ), Heidelberg, Germany; 4 Department of Molecular Medicine, Sapienza University of Rome, Rome, Italy; 5 Institut de Neurophysiopathologie, Aix-Marseille Université, CNRS, Marseille, France; 6 Department of Radiological, Oncological and Anatomo-Pathological Sciences, Sapienza University of Rome, Rome, Italy; 7 Department of Pediatric Hematology and Oncology, Cell and Gene Therapy, IRCCS Bambino Gesù Children’s Hospital, Rome, Italy; 8 Pathology Unit, Department of Laboratories, IRCCS Bambino Gesù Children’s Hospital, Rome, Italy; 9 Department of Women, Children and Public Health Sciences, Policlinico Universitario A. Gemelli, Catholic University Sacro Cuore, Rome, Italy; 10 Pediatric Neurosurgery, Policlinico Universitario A. Gemelli, Catholic University Sacro Cuore, Rome, Italy; 11 Department of Pediatric Hematology and Oncology, Cell and Gene Therapy, IRCCS Bambino Gesù Children’s Hospital, Department of Gynecology/Obstetrics & Pediatrics, Sapienza University of Rome, Rome, Italy; 12 Pediatric Glioma Research Group, German Cancer Research Center (DKFZ), Hopp Children’s Cancer Center Heidelberg (KiTZ), Heidelberg, Germany; 13 Service d’Anatomie Pathologique Et de Neuropathologie, Hôpital de La Timone, Institut de Neurophysiopathologie, Aix-Marseille Université, AP-HM, CNRS, Marseille, France; 14 Division of Pediatric Neurooncology, Hopp Children’s Cancer Center Heidelberg (KiTZ), German Cancer Consortium (DKTK), German Cancer Research Center (DKFZ), and Department of Pediatric Oncology, Hematology and Immunology, Heidelberg University Hospital, Heidelberg, Germany; 15 Department of Radiological, Oncological and Anatomo-Pathological Sciences, IRCCS Neuromed, Pozzilli, Italy

**Keywords:** Pediatric low-grade gliomas, miR-1248, Prognostic biomarker, Tumour progression, Risk stratification, Personalized medicine

## Abstract

**Background:**

Pediatric low-grade gliomas (pLGGs), particularly incompletely resected supratentorial tumours, can undergo progression after surgery. However to date, there are no predictive biomarkers for progression. Here, we aimed to identify pLGG-specific microRNA signatures and evaluate their value as a prognostic tool.

**Methods:**

We identified and validated supratentorial incompletey resected pLGG-specific microRNAs in independent cohorts from four European Pediatric Neuro-Oncology Centres.

**Results:**

These microRNAs demonstrated high accuracy in differentiating patients with or without progression. Specifically, incompletely resected supratentorial pLGGs with disease progression showed significantly higher miR-1248 combined with lower miR-376a-3p and miR-888-5p levels than tumours without progression. A significant (*p* < 0.001) prognostic performance for miR-1248 was reported with an area under the curve (AUC) of 1.00. We also highlighted a critical oncogenic role for miR-1248 in gliomas tumours. Indeed, high miR-1248 levels maintain low its validated target genes (CDKN1A (p21)/FRK/SPOP/VHL/MTAP) and consequently sustain the activation of oncogenic pathways.

**Conclusions:**

Altogether, we provide a novel molecular biomarker able to successfully identify pLGG patients associated with disease progression that could support the clinicians in the decision-making strategy, advancing personalized medicine.

**Supplementary Information:**

The online version contains supplementary material available at 10.1186/s40364-022-00389-x.

## Background

Pediatric low-grade gliomas (pLGGs) are a histologically heterogeneous group of tumours which include pilocytic astrocytomas (PA), the most frequent histology, and non-PA tumours, that comprise angiocentric gliomas (AG), dysembryoplastic neuroepithelial tumours (DNET), gangliogliomas (GG), and glioneuronal tumours (GNT). These tumours are classified as grade 1 and 2 tumours by the WHO and account for 40% of brain tumours under the age of 18 years [1–4].

Safely resectable pLGGs, primarily occurring in the cerebellum and in non-eloquent supratentorial locations, can often be cured by surgery with an excellent long-term prognosis [5]. Deep supratentorial tumours and those arising in the brainstem are more difficult to resect. Of note, disease progression occurs in about 30% of the cases, and these patients receive adjuvant chemotherapy and, in selected cases, radiotherapy (e.g. SIOP‐LGG 2004 protocol and LOGGIC/LOGGIC Core) [6, 7]**.**

The cost of survivorship is often high, since patients frequently present long-term sequelae both by the disease itself and as a consequence of the treatment, such as epilepsy, vision loss, impaired motor skills and cognitive dysfunction [8].

The genomic landscape of pLGGs has allowed the revision of the WHO classification resulting in the new 2021 WHO classification of the tumours of the Central Nervous System (CNS) [4]. Moreover, recent studies have stratified patients into different risk classes on the basis of selected genetic alterations [9] and on histological and clinical variables [10].

Research groups, including ours, have investigated microRNA profiles in pLGGs. Profiling studies on PAs demonstrated the over-expression of miR-21 and miR-34 and the downregulation of miR-124 and miR-129 compared to normal brain tissue [11–14]. Furthermore, miR-487b was reported to be downregulated in these tumours [12, 15, 16]. Low levels of miR-10b-5p were reported in sporadic and NF1-associated PAs [17]. In addition, miR-20a-5p was identified as a regulator of both the MAPK/ERK and mTORC1 pathways, in a subset of genetically driven astrocytomas [18], while miR-125b was under-expressed in pleomorphic xanthoastrocytomas and GG [19]. Finally, miR-519d and miR-4758 were differentially expressed in GG compared to other histological subtypes [20].

In this context, we previously investigated a cohort of supratentorial PA and non-PA tumours, showing that all of them are characterized by low expression of miR-139-5p and that this deregulation contributes to tumour growth by sustaining the pro-tumoral PI3K/AKT signalling pathway [21].

Two studies investigated microRNAs as possible prognostic biomarkers in pLGGs. Low levels of miR-29b-3p together with a high level of its target gene, cannabinoid receptor 1 (CNR1), were suggested to be predictive of residual disease involution in a cohort of subtotally resected pLGGs [22]. In the second study, a 7-microRNAs signature was associated with chemotherapy response [23].

The identification of high risk of progression in incompletely resected pLGGs remains to date an unmet clinical need. Therefore, the aim of this study was to analyse microRNA profiles in pLGG tissue samples in a large cohort of incompletely resected patients to identify potential biomarkers of progression. To this end, samples were collected from independent cohorts of patients from four European Pediatric Neuro-Oncology Centres and two different technologies were used to determine microRNAs able to differentiate patients with and without disease progression. Finally, we validated miR-1248 as a prognostic biomarker for tumours with progression.

## Materials and methods

### Characteristics of pLGG samples

This study was performed on 43 supratentorial incompletely resected pLGG samples (PA and non-PA) collected from four European pediatric neuro-oncology centres. The workflow of the study is depicted in Supplementary Figure S1.

The first cohort (Cohort I) derived from three independent European institutions (Bambino Gesù Children’s Hospital, A. Gemelli Hospital and Marseille Hospital) and consisted of 20 PA and 9 non-PA tumour samples. The second cohort (Cohort II) derived from one European institution, the DKFZ German Research Centre and consisted of 9 PA tumour samples.

According to the extent of surgical resection on Magnetic Resonance Imaging (MRI) after one month post-surgery or post biopsy, patients were subdivided in those with near total resection (NTR), where an amount between 50–90% of the mass was removed, and those with biopsy, where less than 50% of the tumour was removed.

Patients with any residual tumour (NTR and biopsy) were further subdivided in with or without progression according to disease progression, on the basis of MRI evaluation during the follow up and/or clinical recurrence identified by clinicians [1]. Kaplan Meier analysis was performed using the progression free survival (PFS) data reported in Tables 1 and 2, and the results demonstrated a lower PFS for pLGG patients with progression compared to patients without progression (Supplementary Figure S2).

**Table 1:** Clinical-pathological features of Cohort I pLGG patients

Cohort I pLGG patients										
**Sample code**	**Age (years)**	**Gender**	**Site of onset**	**Histology**	**Type of resection**	**Progression**	**Progression Free Survival (years)**	**Overall Survival (years)**	**Mutational status of BRAF**	**Profiling (P) / ddPCR (d)**
22233	5	M	H Supra	DNET	NTR	WITH	2	9	NA	P + d
OPBG13P	4	F	H Supra	DNET	NTR	WITH	4.4	8	WT	P
16526	13	F	H Supra	GG	NTR	WITH	7	8	V600E	P + d
19683	12	F	H Supra	GG	NTR	WITH	2	9	WT*	P + d
22457	9	M	H Supra	GG	NTR	WITH	7	10	V600E	P + d
11364	12	M	H Supra	GG	NTR	WITH	5	10	WT*	P + d
25,595	4	M	H Supra	PA	NTR	WITH	1	NA	WT*	P + d
11720	2	M	H Supra	PA	NTR	WITH	2	NA	WT*	P + d
8380	16	F	H Supra	PA	NTR	WITH	2	12	WT*	P + d
21617	6	M	H Supra	PA	NTR	WITH	5	13	WT*	P + d
256 13	3.6	M	Md Supra	PA	NTR	WITH	0.58	7	K15B9	P + d
OPBG45C	7	F	Md Supra	GG	Biopsy	WITH	4	7	WT	P + d
OPBG62P	3.7	M	Md Supra	GG	Biopsy	WITH	0.5	5	WT	P + d
OPBG51S	6.8	F	Md Supra	PA	NTR	WITH	0.66	5	K16B11	P + d
119637 FF	6	M	Md Supra	PA	NTR	WITH	1.58	14	K16B9	P + d
179435 FF	6	M	Md Supra	PA	NTR	WITH	4.25	17	K16B9	P + d
177408 FF	6	M	Md Supra	PA	NTR	WITH	0.75	12	WT*	P + d
123965 FF	8	F	Md Supra	PA	NTR	WITH	2.08	16	K15B9	P + d
89636 FF	2	F	Md Supra	PA	NTR	WITH	9.33	25	WT*	P
117945 FF	0.91	M	Md Supra	PA	NTR	WITH	1.83	17	WT	P + d
OPBG135F	7	M	Md Supra	PA	Biopsy	WITH	0.5	1.66	WT*	d
1651	13	M	H Supra	AG	NTR	W/O	8	8	WT*	P + d
OPBG54M	8	F	H Supra	DNET	NTR	W/O	5	5	WT	P + d
OPBG74M	5	F	H Supra	DNET	NTR	W/O	2.16	2.16	WT*	P + d
OPBG112I	14	F	H Supra	GG	NTR	W/O	0.33	0.33	V600E	d
OPBG94C	5.3	F	Md Supra	PA	NTR	W/O	1	1	WT*	P + d
OPBG43D	3	M	Md Supra	PA	NTR	W/O	3.75	3.75	K16B9	P + d
OPBG58SP	5	M	Md Supra	PA	NTR	W/O	3	3	WT	P + d
172524 FF	14	M	Md Supra	PA	NTR	W/O	12	12	WT*	P + d
45723 FF	16	F	Md Supra	PA	NTR	W/O	17	17	V600E	P + d
75683 FF	7	M	Md Supra	PA	NTR	W/O	16	16	WT*	P + d
OPBG99D	10	M	Md Supra	GNT	NTR	W/O	0.91	0.91	V600E	d
OPBG115C	7	F	Md Supra	PA	Biopsy	W/O	0.33	0.33	K15B9	d
OPBG117C	10	F	Md Supra	GG	Biopsy	W/O	0.5	0.5	WT	d

*H* Hemispheric, *M* Midline, *DNET* Dysembryoplastic Neuropepithelial Tumor, *GG* Ganglioglioma, *GT* Glioneuronal Tumor, *PA* Pilocytic Astrocytoma, *AG* Angiocentric Glioma, *NTR* Near Total Resection, *WITH* With progression, *W/O* Without progression, *CR* Complete remission, *DoD* Dead of Disease, *SD* Stable Disease, *PR* Partial Remission, *NA* Not applicable, *ND* Not detected. BRAF screening was limited to the V600E point mutation and three fusion genes [KIAA1549-BRAF exon 16-exon 9 (K16B9), KIAA1549-BRAF exon 16-exon 11 (K16B11), KIAA1549-BRAF exon 15-exon 9 (K15B9)]. WT* = Not screened for BRAF K15B9

**Table 2:** Clinical-pathological features of Cohort II pLGG patients

Cohort II pLGG patients									
**Sample code**	**Age (years)**	**Gender**	**Site of onset**	**Histology**	**Type of resection**	**Progression**	**Progression Free Survival (months)**	**Overall Survival (months)**	**Mutational status**
ICGC_PA104	3	F	Md Supra	PA	NTR	W/O	6	6	WT
ICGC_PA144	2	F	Md Supra	PA	NTR	W/O	4	4	CLCN6:BRAF Ex2:Ex11); BRAF p.E451D
ICGC_PA14	5	F	Md Supra	PA	NTR	W/O	23	23	K15B9
ICGC_PA159	5	M	Md Supra	PA	NTR	W/O	3	3	QKI:NTRK2 (Ex6:Ex16)
ICGC_PA4	1	M	Md Supra	PA	NTR	W/O	22	22	K16B9
ICGC_PA54	4	F	Md Supra	PA	NTR	WITH	4	12	WT
ICGC_PA69	6	F	Md Supra	PA	NTR	WITH	17	17	NF1 Large deletion (somatic); p.Q1174fs (somatic); FGFR1 p.N546K
ICGC_PA71	2	F	Md Supra	PA	NTR	WITH	5	16	NF1 p.Q959X (germline); large deletion (somatic)
ICGC_PA84	9	M	Md Supra	PA	NTR	W/O	10	10	FGFR1 p.K656E; PTPN11 p.E76A

*Md* Midline, *PA* Pilocytic Astrocytoma, *NTR* Near Total Resection, *WITH* With progression, *W/O* Without progression

Histological diagnosis was performed according to WHO 2021 classification criteria [4] by experienced neuropathologists (FG, FDC, DFB). All patients before surgery were naïve for chemo and/or radiotherapy. Ethical approval (Rif. 5866) was obtained in accordance with the Helsinki declaration of 1964 and its later amendments. Informed written consent was obtained from the patients, parents or guardians before enrolment, according to our ethical committee guidelines.

### Samples used for microRNA profiling

MicroRNA expression levels were obtained from 29 supratentorial Grade I PA and non-PA tumours belonging to Cohort I by the use of Real-Time quantitative PCR (RT-qPCR) TaqMan Low Density Array (TLDA) microfluidic cards (Human miR v3.0, Life Technologies). Clinical and pathological data of Cohort I are reported in Table 1. Mean age was 7.6 years, with a median of 7. The most frequent histology was PA (*n* = 20, 59%), followed by GG (*n* = 8, 23%), DNET (*n* = 4, 12%), AG (*n* = 1, 3%) and glioneuronal tumour (GNT, *n* = 1, 3%). Sixteen PAs (80%) were located in midline structures: six in optic pathways, five in the third ventricle and four in the thalamus. Only four (20%) arose in hemispheric structures. DNET and AG were hemispheric, while the GNT arose in the third ventricle. Five GG arose in the temporal lobe, while three in midline structures, specifically one in the diencephalon, one in optic pathways and one in thalamus. (62%, DNET *n* = 2; GG *n *= 6; PA *n* = 13) experienced tumour progression with a PFS average time of 4.5 years and tumours were equally distributed between hemispheric (*n* = 10) and midline (*n* = 11) areas. Thirteen patients (38%, AG *n* = 1; DNET *n* = 2; GG *n* = 2; GNT *n* = 1; PA *n* = 6) did not progress, with tumours mostly located in midline structures (9 out of 13, 69%). Of note, DNA methylation analysis was performed in 18 samples (Supplementary Table S1). On the basis of the new classifier version (v12.3) [24, 25] high score (≥ 0.84) was obtained in 12/18 (67%) patients and histological diagnosis was confirmed in 9/12 (75%) cases. Conversely, three tumours with an original pathological diagnosis of GG were classified as PA. 6/18 (33%) tumours were attributed a low methylation score with consequent inconsistent methylation classification.

MicroRNA-sequencing was performed in the 9 samples of Cohort II at the DKFZ German Research Centre to obtain microRNA expression profiles. Clinical and pathological features of this cohort are reported in Table 2. All patients underwent NTR and the mean age at diagnosis was 4.1, while the median age was 5 years old. All NTR supratentorial tumours involved midline structures: six were located in the optic pathways (60%), two in the thalamus (20%) and two in the third ventricle (20%). In the NTR subgroup, three patients (33.3%) presented progression with a mean PFS of 8.6 months.

### Samples used for validation of profiling results

Validation of profiling results was performed in a series of Cohort I samples (27 out of the 29 that were used for profiling plus 5 new samples) through droplet digital PCR (ddPCR). Clinical and pathological features of samples are reported in Table 1, indicating which samples were used for profiling and validation of the results.

### Genomic landscape of samples

Cohort I samples were screened for the KIAA1549:BRAF fusion gene variants (KIAA1549:BRAF exon16-exon9 (K16B9) (*n* = 33) and KIAA1549:BRAF exon16-exon11 (K16B11) (*n* = 33)) using the Applied Biosystems ViiA 7 RT-qPCR system and validated by PCR-based Sanger sequencing. The latter was also used for the detection of KIAA1549:BRAF exon15-exon9 (K15B9) fusion (*n* = 19). The BRAF V600E point mutation (*n* = 33) was analysed by RT-qPCR using TaqMan probes, as previously described [21] (Table 1). DNA methylation of Cohort I samples (*n* = 18) was also evaluated by DNA Methylation array [21]. Results were analysed both with the old (v11b4) and new (v12.3) classifier versions (Supplementary Table S1). Samples with calibrated scores higher than 0.84 were considered informative, while low scores suggesting classifier prediction uncertainty were not taken into consideration [26]. In addition to microRNA expression levels both RNA sequencing and whole genome sequencing data were available for seven samples of Cohort II (Table 2) [27].

### MicroRNA profiles

For Cohort I, microRNA expression profiling was performed on fresh frozen (FF) (*n* = 19) or FFPE (*n* = 9) tissues using TLDA microfluidic cards (Human miR v3.0, Life Technologies), that analyse the 754 most studied microRNAs, as previously reported [21]. For Cohort II samples microRNA sequencing, that allows the assessment of all known microRNAs (2656 mature microRNAs in the latest release of miRBase; http://www.mirbase.org/) [28], was performed in the DKFZ German Research Centre Heidelberg, as previously described [29].

### Droplet Digital PCR (ddPCR)

Ten nanograms of RNA were retrotranscribed using TaqMan™ MicroRNA Assay (Life Technologies) according to manufacturer’s instructions. The resulting cDNA was diluted 1:6 and 8 μl were used to prepare a 22 μl reaction mix containing 11 μl of 2X ddPCR Supermix for Probes (Bio-Rad) and 1,1 μl 20X TaqMan miRNA PCR primer probe set (Life Technologies). The PCR mixes for each sample were loaded in a disposable cartridge (Bio-Rad) together with 70 μl of droplet generation Oil (Bio-Rad) and loaded in the QX200 droplet generator (Bio-Rad).

40 μl of droplets were then transferred in a 96 well plate and an endpoint PCR was performed using the following conditions: 95 °C for 10 min, then 45 cycles of 95 °C for 15 s and 58 °C for 1 min, and a final step at 98 °C for 10 min. Then, the 96 well plate was placed in the QX200 Droplet Reader for detection of positive droplets. The quantification of positive droplets was performed using the QuantaSoft software (Bio-Rad).

### Statistical analysis

MicroRNA expression profiling data from Cohort I were processed with Statminer™ Software v 5.0 (Integromics TM) and differential expression analysis between pLGG samples with and without progression was performed, as previously described [21]. Briefly, microRNA expression was normalized using the global normalization and differential expression was evaluated using the limma test. *P* values < 0.05 were considered as statistically significant.

MicroRNA sequencing data of Cohort II were analysed as described in [29]. Samples were classified based on the presence or absence of progression, and differential expression analysis was performed using the R package DESeq2. *P* values < 0.05 were considered statistically significant.

Clustering and heatmaps were also generated in R using the pheatmap function. The different range of microRNA expression values in the two cohorts (Cycle Threshold and Reads per Million mapped reads values), is represented by the diverse heatmap colours. Kaplan Meier analyses were performed using PFS and OS data of all patients from cohorts I and II using GraphPad Prism software Version 8 (La Jolla, CA).

For ddPCR data, statistical analyses were performed using GraphPad Prism software Version 8 (La Jolla, CA). The t-test for unpaired data was used to analyse differences in microRNA expression between experimental groups. *P* values < 0.05 were considered statistically significant.

Receiver operating characteristic (ROC) curves for the microRNAs of interest were calculated using GraphPad Prism version 8 (La Jolla, California, USA).

Univariate analysis was performed in IBM SPSS Statistics version 27 (Armonk, New York, USA), using the General Linear Model (GLM) for miR-1248. In detail, the Progression Free Survival (PFS) was used as a covariate and other clinical features (site of onset, histology, BRAF status, age, sex and type of resection) as fixed factors. Univariate analysis was performed to determine the significance of the clinical features on the expression of the microRNA. The Parameter Estimates summarized the effect of each clinical factor. P < 0.05 was considered statistically significant.

MicroRNA validated target identification was performed for miR-1248 downloading the validated targets genes for Homo sapiens Release 8.0 from miRTarBase [30]. Tumour suppressor genes were downloaded from TSGene 2.0 web tool [31], that reports 983 downregulated tumour suppressor genes in The Cancer Genome Atlas (TCGA) pan-cancer samples compared to normal samples. Intersection of validated target genes of miR-1248 with 983 tumour suppressor genes was performed.

## Results

### Cohort I microRNA array profiling

**Fig. 1:**
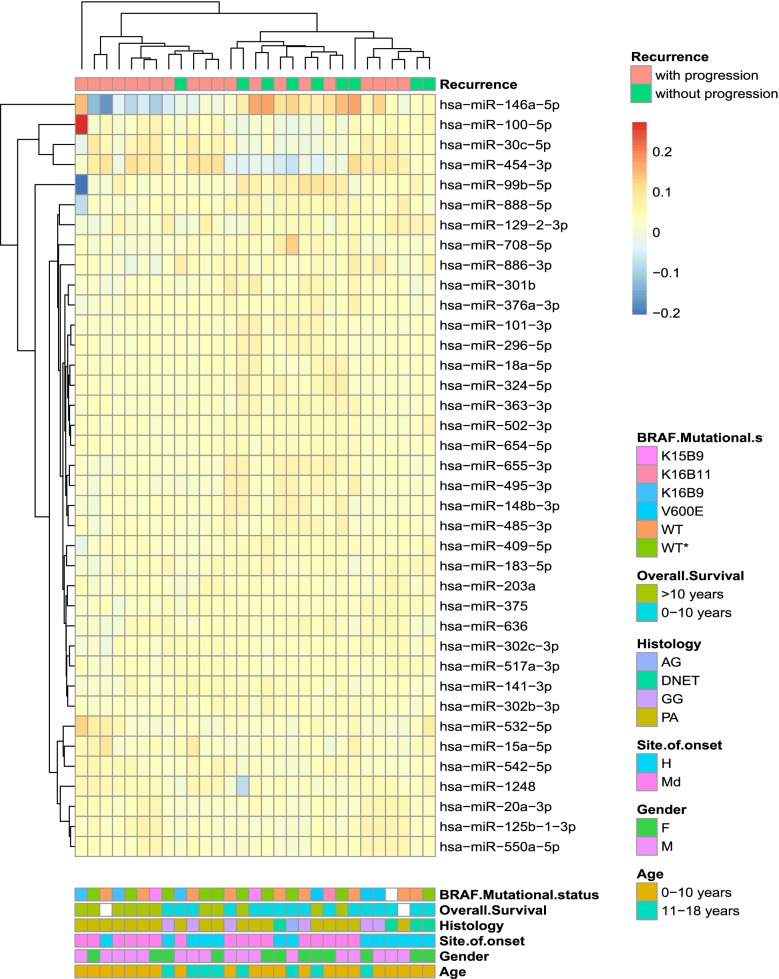
Hierarchical clustering of microRNAs displaying differential expression in supratentorial pLGGs with progression (pink) vs. pLGG without progression (green) belonging to the first cohort (Cohort I). Hierarchical clustering of the 26 microRNAs differentially expressed in pLGGs with progression (*n* = 20) vs. pLGG without progression (*n* = 9) and 12 microRNAs amplified in one group (*p* < 0.05) was performed and the bray method was used to generate clusters on the basis of delta cycle threshold values

MicroRNA profiling was performed on supratentorial PA and non-PA samples from Cohort I (Table 1) divided in tumours with or without progression (see materials and methods for details). We found a higher number of detectable microRNAs in the subgroup of patients without progression (401/754, 53%) in comparison to those with progression (331/754, 44%). Differential expression analysis resulted in 38 microRNAs, nine upregulated and 29 downregulated in tumours with progression (Supplementary Table S2). Hierarchical clustering distinguished two subgroups: one branch included predominantly tumours with progression and the second branch mixed samples (Fig. 1).

All features of the samples included in the study are reported in Fig. 1 and Table 1.

Of note, samples with progression were equally distributed between hemispheric (*n* = 10) and midline (*n* = 10) areas, whereas those who did not progress were all located in midline structures (*n* = 9). This result underlines that differentially expressed (DE) microRNAs reflect at least in part the distinct embryologic origin between tumours, hemispheric or midline, respectively.

### Cohort II microRNA sequencing

**Fig. 2:**
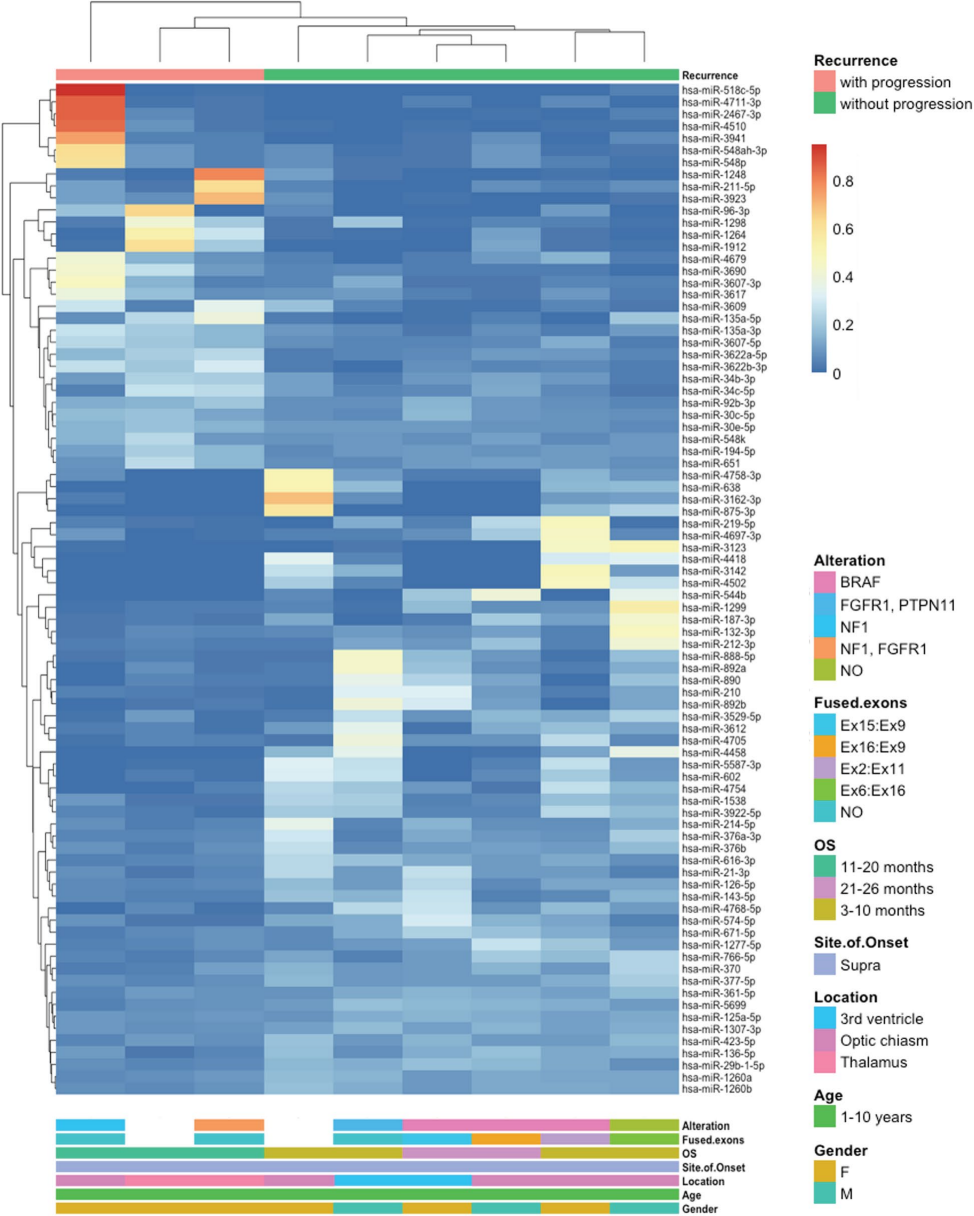
Hierarchical clustering of microRNAs displaying differential expression in supratentorial PA with progression (pink) vs. supratentorial PA without progression (green) belonging to the second cohort (Cohort II). Hierarchical clustering of the 84 microRNAs differentially expressed in supratentorial PA with progression (*n* = 3) *vs.* supratentorial PA (*n* = 6) without progression (*p* < 0.05) was performed and the bray method was used to generate clusters on the basis of reads per million mapped reads (RPM) values

As for Cohort I, a second independent cohort included supratentorial PA samples subdivided into tumours with or without progression (Table 2). The microRNA differential expression analysis resulted in 32 upregulated and 52 downregulated microRNAs in tumours with progression (Supplementary Tables S3). The DE microRNAs were used for hierarchical clustering analysis, which showed distinct branches for tumours that progressed from the ones that did not (Fig. 2).

All features of the samples included in the study are reported in Fig. 2 and Table 2. Samples with progression were located in the thalamus (*n* = 2) and in the optic pathway (*n* = 1), while tumours that did not progress arose in the optic pathway (*n *= 4) and in the third ventricle (*n* = 2).

### Deregulated microRNAs in the two cohorts

**Fig. 3:**
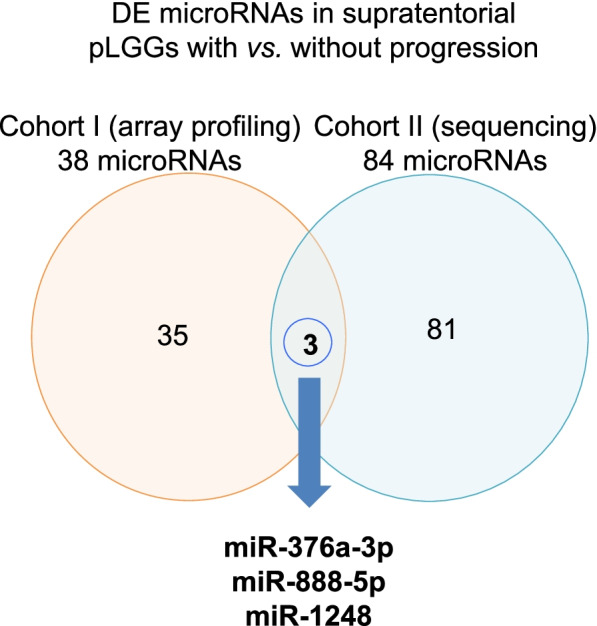
Identification of microRNAs as prognostic biomarkers. Venn diagram of differentially expressed (DE) microRNAs in supratentorial pLGGs with vs. without progression (Cohort I in orange, Cohort II in blue)

Since our aim was to identify microRNAs able to stratify incompletely resected pLGG patients into progression risk categories, we further analysed the results obtained from Cohort I and II focusing on the deregulated microRNAs in both cohorts. Specifically, analysing data generated from two independent cohorts with two different technologies and considering all samples from both cohorts, we were able to identify three deregulated microRNAs. In detail, miR-376a-3p and miR-888-5p were downregulated, while miR-1248 was upregulated in pLGGs, both PA and non-PA, with progression (Fig. 3 and Supplementary Figure S3).

### Evaluation of miR-376a-3p, miR-888-5p and miR-1248 as prognostic biomarkers

With the aim to validate the profiling results, miR-376a-3p, miR-888-5p and miR-1248 expression levels were evaluated using an absolute quantification method. Specifically, the three microRNAs were analysed in 27 out of 29 samples previously used for profiling plus 5 new cases for a total of 32 samples (see Table 1).

**Fig.4:**
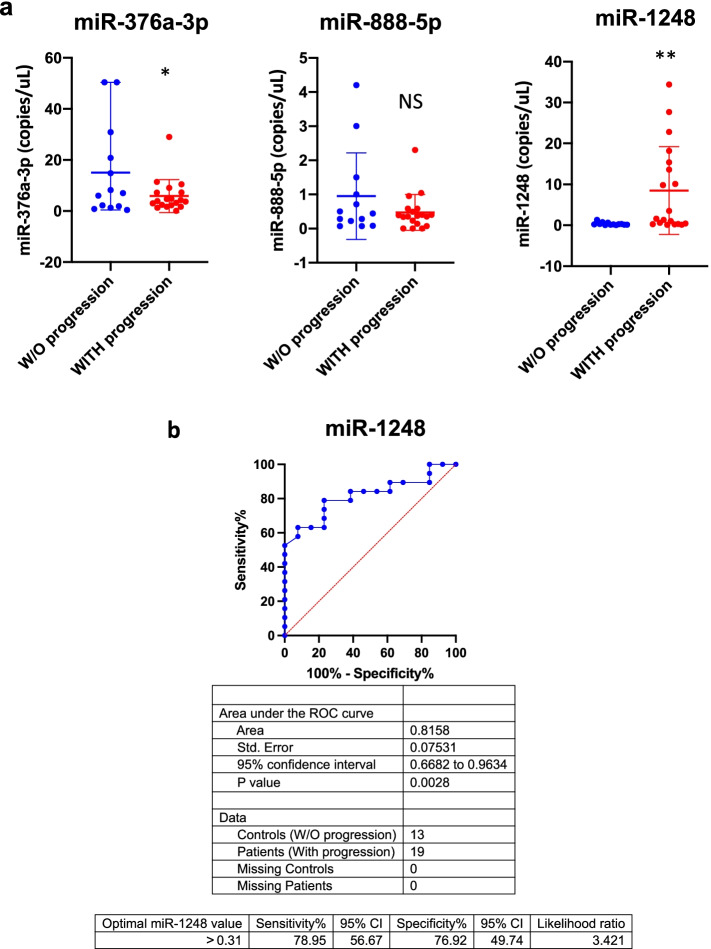
Three microRNAs deregulated in incompletely resected pLGGs. **a** ddPCR copies/μL of miR-376a-3p (left), miR-888-5p (middle) and miR-1248 (right) in supratentorial pLGGs without (W/O, blue dots) vs. with progression (red dots). * *p* < 0.05, ** *p* < 0.01, *** *p* < 0.001, **** *p* < 0.0001. **b** ROC curve of miR-1248 (AUC = 0.8158, *p* = 0.0028). Blue line = sensitivity, red line = identity

DdPCR allowed to confirm the downregulation of miR-376a-3p and upregulation of miR-1248 in both PA and non-PA tumours with progression, whereas miR-888-5p was not statistically significant (Fig. 4a).

ROC analysis was performed to test the specificity and sensitivity of expression of these microRNAs. MiR-376a-3p (Supplementary Figure S4) was not statistically significant, while miR-1248 resulted in an AUC = 0.815 (95% CI: 0.6682 to 0.9634) and a P value of 0.0028 (optimal miR-1248 cut-off value for pLGGs with progression > 0.31 copies/μL) (Fig. 4b). This result highlights that miR-1248 is able to distinguish both PA and non-PA with progression respect to those without.

Next, we proceeded with *in-silico* analysis in order to identify potential targets of miR-1248. First, miR-1248 validated target genes were queried and 180 genes were obtained. Next, we compared the 180 miR-1248 validated target genes with tumour suppressor genes downregulated in TCGA cancer samples compared to normal tissue samples. We identified 10 miR-1248 validated target genes (Table 3) and literature data confirmed the oncosuppressive role for five of them in pediatric and/or adult gliomas, namely Cyclin-dependent Kinase Inhibitor 1A (CDKN1A), Fyn-related SRC Family Tyrosine kinase (FRK), S-methyl-5'-thioadenosine phosphorylase (MTAP), Speckle-type POZ protein (SPOP) and von Hippel-Lindau disease tumour suppressor (VHL). Of interest, this can potentially explain the oncogenic properties of this microRNA in the context of pLGGs.

**Table 3:** Overview of the 10 miR-1248 validated target genes included in the TSGene 2.0 database

Official symbol	Official full name	Sequence accession ID	Function
ANAPC1	Anaphase promoting complex subunit 1	NM_022662.4	ANAPC1 is a component of the anaphase-promoting complex, an E3 ubiquitin ligase complex that controls progression through the metaphase to anaphase of the cell cycle
CDKN1A	Cyclin-dependent Kinase Inhibitor 1A	NM_000389.4	P21 acts as a regulator of cell cycle progression at G1 in a p53-dependent and independent way. P21 plays regulatory roles in S phase DNA replication and DNA damage repair
FRK	Fyn-related SRC Family Tyrosine kinase	NM_002031.2	FRK suppresses cell growth and promotes PTEN protein stability. FRK may function as a tumour suppressor protein
HIVEP3	HIVEP zinc-finger 3	NM_001127714.3	HIVEP3 is a transcription factor that inhibits TNF-α induced NF-κB activation. HIVEP3 may interacts with TRAF proteins inhibiting the c-Jun/JNK signalling pathway
MTAP	S-methyl-5'-thioadenosine phosphorylase	NM_002451.3	MTAP is an enzyme involved in polyamine metabolism and its down-regulation and/or deletion has been described in many cancers
NOTCH2	Notch receptor 2	NM_001200001.2	NOTCH2 belongs to an evolutionarily conserved intercellular signalling pathway with roles in cell fate decisions, such proliferation, differentiation and apoptosis
PRKCB	Protein kinase C beta	NM_002738.7	PRKCB is a serine/threonine protein kinase involved in several cellular processes, such as oxidative stress induced apoptosis, insulin signalling and glucose transport regulation
SH2B3	SH2B adaptor protein 3	NM_001291424.1	SH2B3 is a negative regulator of cytokine signalling with critical roles in hematopoiesis
SPOP	Speckle-type POZ protein	NM_001007226.1	SPOP is a member of the E3 ubiquitin-protein ligase complex and mediates the ubiquitination and subsequent degradation of target proteins
VHL	von Hippel-Lindau disease tumor suppressor	NM_000551.3	VHL is a member of a protein complex with ubiquitin ligase E3 activity, being involved in the ubiquitination and degradation of hypoxia-inducible factors

### MiR-1248 univariate analysis

Univariate analysis was performed for miR-1248 using ddPCR data to evaluate whether it could be associated with the other clinical features of PA and non-PA patients described in Table 1. The parameter estimates resulted in statistically significant results for two clinical features, site of onset and histology (Supplementary Table S4), while the association of miR-1248 expression with patient age, sex, extent of resection, histology and BRAF status was not statistically significant.

Considering the site of onset, the observed power of effect of hemispheric site of location for miR-1248 was 0.969, whereas the effect of DNET histology was 0.987. However, the number of DNET samples evaluated in ddPCR was too small, therefore we focused our attention on the hemispheric site of location and its association with miR-1248.

### MiR-1248 prognostic biomarker of progressive supratentorial hemispheric pLGGs

Based on the univariate analysis results and considering that all analysed pLGG, PA and non-PA, with progression included two distinct sites of onset, midline and hemispheric, we further analysed miR-1248 expression levels subgrouping samples into these two categories.

**Fig. 5:**
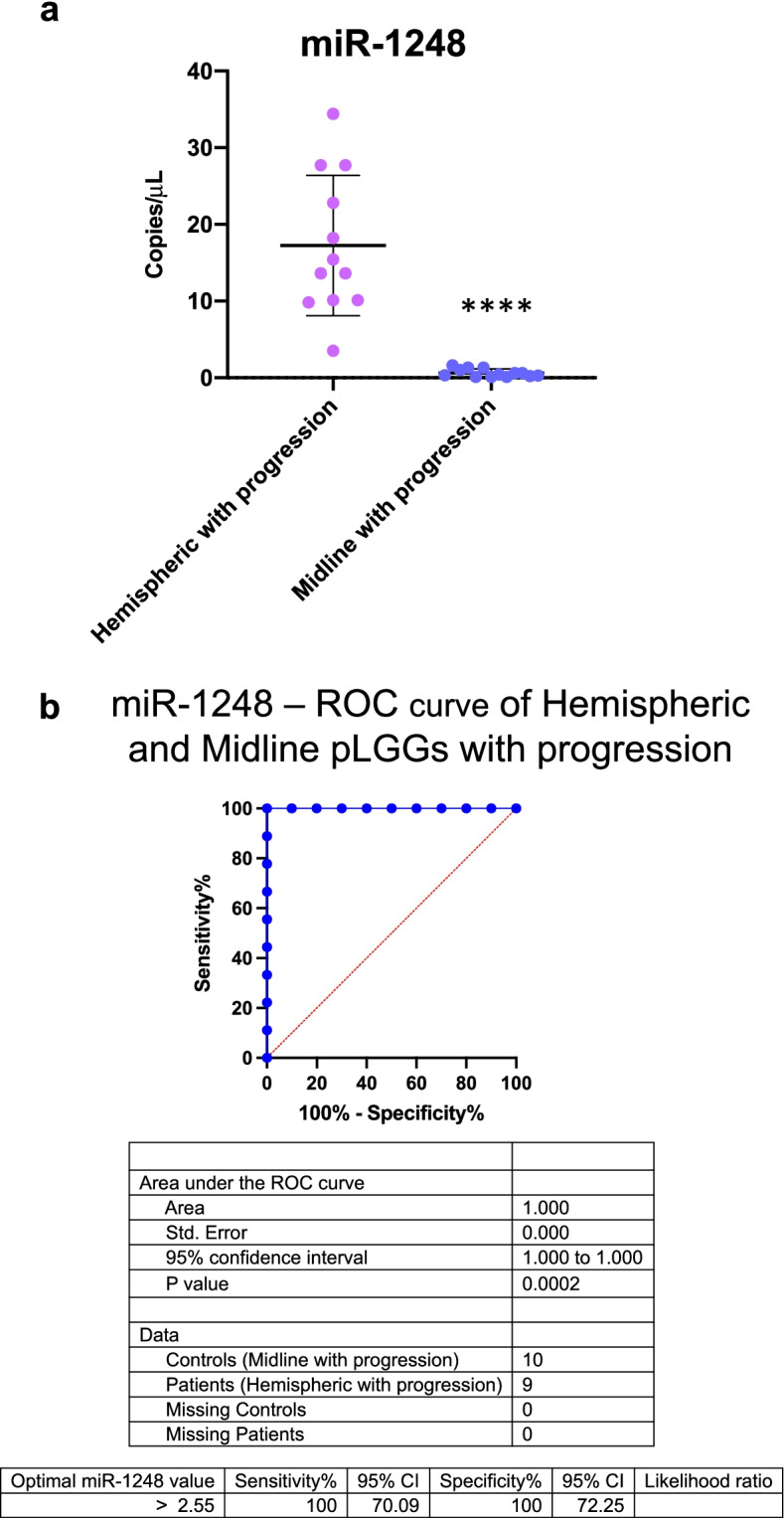
MiR-1248 as prognostic biomarker for hemispheric pLGGs. **a** ddPCR copies/μL of miR-1248 in hemispheric (lavender dots) vs. midline (blue dots) pLGGs with progression. * *p* < 0.05, ** *p* < 0.01, *** *p* < 0.001, **** *p* < 0.0001 (**b**) ROC curve of miR-1248 in hemispheric and midline pLGGs with progression (AUC = 1.00; p-value = 0.0002). Blue line = sensitivity, red line = identity

Notably, miR-1248 was upregulated only in hemispheric tumours in comparison with midline tumours (Fig. 5a) and this differential expression was maintained when examining all the analysed histologies (Supplementary Figure S5). Moreover, ROC analysis was performed for miR-1248 in hemispheric and midline pLGG tumours with progression and highlighted the high performance of miR-1248 as prognostic biomarker in hemispheric pLGGs with progression (AUC = 1.00, 95% CI: 1.00 to 1.00; *P* value = 0.0002; optimal miR-1248 cut-off value for hemispheric pLGGs with progression > 2.55 copies/uL) (Fig. 5b). These results illustrate that by combining the site of onset with the expression level of miR-1248 it is possible to identify tumours with higher risk of progression after incomplete surgical resection.

### Network depicting the oncogenic role of miR-1248 with its validated target genes in gliomas

**Fig. 6:**
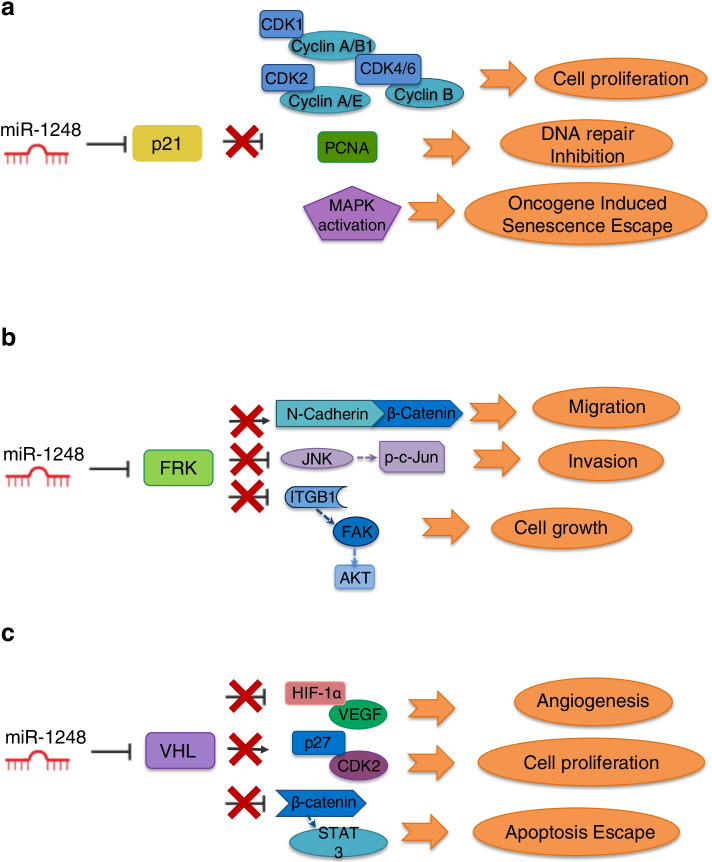
Schematic model of miR-1248 oncogenic role in pLGGs. **a** p21 inhibition by miR-1248 leads to cell proliferation, impaired DNA repair and escape from oncogene induced senescence. **b** miR-1248 maintains low FRK levels promoting migration, invasion and growth. **c** miR-1248 targeting of VHL contributes to angiogenesis, cell proliferation and escape from the apoptotic program

Taking into consideration the information provided by other studies on glioma tumours, we propose a model (Fig. 6) recapitulating miR-1248 possible biological role in pLGGs. Indeed, the oncogenic role of miR-1248 in pLGGs emerges by examining its previously mentioned validated targets. The first, CDKN1A (p21), is a master senescence marker thus acting as a tumour suppressor [32]. Of note, pLGGs are characterized by oncogene-induced senescence (OIS), which is associated with growth arrest and tumour progression restriction [33]. In addition, Bongaarts et al. described low levels of p21 in a cohort of adult and pediatric GG, however they did not take into consideration the extent of resection nor the tumour progression [20]. Therefore, the high levels of miR-1248 in supratentorial hemispheric tumours with progression may in part reflect the loss of p21 expression in this subgroup of pLGGs. The activity of other miR-1248 validated oncosuppressor target genes, namely FRK, VHL, SPOP and MTAP, has been described by different studies. The oncosuppressor role of FRK has been reported in adult gliomas [34–36]. FRK inhibits glioma cell migration and invasion by interacting with the N-cadherin/β-catenin and with the JNK/c-Jun signalling pathways [34, 36]. Moreover, Wang et al. recently demonstrated that FRK inhibits the activity of the Integrin subunit β1 (ITGB1), an upstream regulator of AKT, whose functional role in pLGG has been extensively described [21, 37–39]. VHL is involved in HIF-1-alpha degradation, β-catenin/Tcf-4 signalling pathway and AKT activity inhibition therefore exerting a pivotal role as tumour suppressor [40–43]. MTAP and SPOP activity as tumour suppressors is less clear. In detail, SPOP downregulation in adult gliomas has been associated with disease progression and has been positively correlated with mean tumour diameter, tumour grade and histological type [44]. MTAP expression instead has been analysed in a cohort of pediatric and adult PA samples and resulted lost in tumours arisen in the cerebral hemispheres [45]. In addition, MTAP is located on the 9p21 chromosomal region and its loss, together with that of CDKN2A, seems to be correlated with the malignant transformation of pLGGs [46]. Altogether, these studies summarize how high levels of miR-1248 can modulate the expression of tumour suppressor genes promoting the aberrant activation of pathways that lead to cancer maintenance and progression.

## Discussion

pLGGs are ideally treated by complete surgical resection. However, if not feasible, patients in clinical and/or radiological progression receive chemotherapy and, in case of older children, radiotherapy, with a PFS rate in the range of 30–40% after 5 years [47, 48]. Residual disease and/or treatments cause significant morbidity [49]. Recent studies investigated genetic alterations proposing stratification of PA and non-PA patients into risk categories and underlining the need for multiple molecular biomarkers to better define the clinical management of these tumours [9, 10].

In this scenario, our study focused on the search for epigenetic prognostic biomarkers able to predict tumour progression in the absence of a complete surgical resection. Specifically, we focused on microRNAs since they act as pivotal players in gene regulation and are deregulated in cancer [50]. Additionally, microRNAs present a tissue specificity that may be useful to identify different tumour types and their tissue of origin [51], therefore acting as potential diagnostic and prognostic biomarkers [52].

We conducted microRNA analyses with two highly sensitive technologies using samples collected from four different neuro-oncology centres. Despite the different histology and site of onset of the tumour samples, interestingly we were able to identify three deregulated microRNAs and a new prognostic biomarker for supratentorial hemispheric pLGGs with progression. In order to corroborate these results we employed a third highly sensitive and precise method of detection. This further validation on an extended patients’ cohort strengthens the value of miR-1248 as a prognostic biomarker in incompletely resected supratentorial hemispheric pLGGs. Interestingly, its differential expression was also significant when miR-1248 was evaluated on the basis of histology. Indeed, miR-1248 was upregulated not only in supratentorial hemispheric PA with progression compared to supratentorial midline PA with progression, but also in supratentorial hemispheric non-PA with progression when compared to midline supratentorial non-PA with progression. Therefore, we demonstrated that high levels of miR-1248 combined with the site of onset can be used as a tool to classify tumours with high risk of progression after incomplete surgical resection.

A focused literature overview allowed us to hypothesize the way in which miR-1248 exerts its oncogenic role in gliomas (Fig. 6). Some of miR-1248 validated target genes indeed act as key oncosuppressor players in gliomas, therefore low levels of these genes may substantially contribute to the major aggressive behavior of incompletely resected pLGGs with progressive disease in respect to those without progression.

Additionally, a wider study of the literature showed that miR-1248 has already been described as an oncomiR correlated with cancer progression in tumours of different tissue-origin [53–60].

High levels of miR-1248 and its target gene AGTR1 were reported in osteosarcoma (OS) and associated with chemoresistance of OS cells and poor survival of patients [53]. In esophageal squamous cell carcinoma (ESCC), miR-1248 decreased expression by UPK1A antisense RNA 1 lncRNA sponging resulted in suppressed cellular proliferation, migration and invasion, further indicating its oncogenic role [55]. A similar tumorigenic function of miR-1248 was reported in laryngeal squamous cell carcinoma (LSCC) where low expression of circular RNA hsa_circ_0036722 could not sponge miR-1248, thus promoting proliferation by inhibiting the tumour suppressor gene RHCG [56]. High expression levels of miR-1248 inhibited the expression of complement anaphylatoxin C3A in lung squamous carcinoma (LSC), preventing tumour growth and reducing its aggressiveness [57]. In addition, comparison of prostate cancer patients with and without lymphatic dissemination resulted in the identification of an 18-microRNA signature associated with lymphatic dissemination that included the highly expressed miR-1248 [58].

Finally, miR-1248 has also been suggested as a prognostic biomarker in patients with Wilms tumour and ESCC [59, 60], conversely its role as prognostic biomarkers for progressive supratentorial hemispheric pLGGs emerges for the first time in this study.

Altogether these evidences underline the role of miR-1248 as prognostic biomarker for all the incompletely resected supratentorial hemispheric pLGGs, filling the existing void of reliable prognostic biomarkers for patient stratification and offering a tool to guide clinicians' choices for the best treatment strategy. In conclusion, although additional research with functional experiments is required to shed light on the biological activity of miR-1248, its use as prognostic biomarker in combination with tumour location, may help clinicians’ in the decision-making strategy.

## Conclusions

Incomplete surgical resection in pediatric low-grade glioma patients entails repeated cycles of treatment, often with lifelong clinical sequelae and sometimes mortality. The lack of prognostic biomarkers provides a disadvantage for patient management. This work reports for the first time microRNA expression patterns of incompletely resected pediatric-low grade gliomas, with miR-1248 high expression levels observed in patients with progression. Further validation of these results indicated how the use of miR-1248 expression levels along with the anatomic location can be applied for the risk stratification of incompletely resected supratentorial pediatric-low grade glioma patients.

## Supplementary Information

**Additional file 1:** **Figure S1.** Overview of the methodological workflow of the study for the identification of miR-1248 as a progression risk stratification biomarker in pLGGs. **Figure S2.** Progression free survival (PFS) data for pLGG patients of cohorts I and II (*p*-value<0.001). **Figure S3.** (a) Normalized relative Ct expression levels of miR-376a-3p in pLGG without and with progression samples from cohort I (*p-value=0.025). (b) Log2 expression levels of miR-376a-3p in PA without and with progression samples from cohort II (*p-value=0.028). (c) Normalized relative Ct expression levels of miR-888-5p in pLGG without and with progression samples from cohort I (*p-value=0.007). (d) Log2 expression levels of miR-888-5p in PA without and with progression samples from cohort II (*p-value=0.007). (e) Normalized relative Ct expression levels of miR-1248 in pLGG without and with progression samples from cohort I (*p-value= 0.029). (f) Log2 expression levels of miR-1248 in PA without and with progression samples from cohort II (*p-value=0.0119). **Figure S4.** ROC curve of miR-376a-3p in pLGG with and without progression (AUC=0.5891; *p*-value=0.3986). Blue line=sensitivity, red line=identity. **Figure S5.** MiR-1248 levels distinguish Hemispheric PA and non-PA tumours with progression from Midline ones. (a) ddPCR copies/μL of miR-1248 in all the pLGG subgroups with progression. * *p*<0.05 *vs* PA Hemispheric with progression, ° *p*<0.05 *vs* GG Hemispheric with progression (b) ddPCR copies/μL of miR-1248 in Hemispheric PA with progression vs Midline PA with progression. ** *p*<0.01 *vs* PA Hemispheric with progression (c) ddPCR copies/μL of miR-1248 in Hemispheric non-PA with progression vs Midline non-PA with progression. The blue dots refer to hemispheric GG with progression, the fuchsia dot refers to the hemispheric DNET with progression, the turquoise dots refer to the midline GG with progression. * *p*<0.05 *vs* non-PA Hemispheric with progression.

**Additional file 2:** **Table S1.** Methylation profiling of 18 Cohort I samples. **Table S2.** MicroRNAs displaying significantly upregulated and downregulated expression in supratentorial PA and non-PA tumours with and without progression (Cohort I). **Table S3.** MicroRNAs displaying significantly upregulated and downregulated expression in supratentorial PAs with and without progression (Cohort II). **Table S4.** Univariate analyses of miR-1248 in pLGG in pLGG samples of ddPCR.

## Data Availability

The datasets supporting the conclusions of this article are included within the article and its supplementary information files.

## Abbreviations

pLGGs: Pediatric low-grade gliomas
GNTs: Glioneuronal tumours
PA: Pilocytic astrocytoma
CNS: Central nervous system
GG: Gangliogliomas
AG: Angiocentric gliomas
DNET: Dysembryoplastic neuroepithelial tumours
NTR: Near total resection
MRI: Magnetic Resonance Imaging
PFS: Progression Free Survival
TLDA: TaqMan Low Density Array
ddPCR: Droplet Digital PCR
