# Supplementary material for: MiR-1248: a new prognostic biomarker able to identify supratentorial hemispheric pediatric low-grade gliomas patients associated with progression
Source: Biomark Res. 2022 Jun 17;10:44. doi: 10.1186/s40364-022-00389-x (PMC9205050; doi:10.1186/s40364-022-00389-x)
Supplement: Supplementary file 1 — Additional file 1: Figure S1. Overview of the methodological workflow of the study for the identification of miR-1248 as a progression risk stratification biomarker in pLGGs. Figure S2. Progression free survival (PFS) data for pLGG patients of cohorts I and II (p-value<0.001). Figure S3. (a) Normalized relative Ct expression levels of miR-376a-3p in pLGG without and with progression samples from cohort I (*p-value=0.025). (b) Log2 expression levels of miR-376a-3p in PA without and with progression samples from cohort II (*p-value=0.028). (c) Normalized relative Ct expression levels of miR-888-5p in pLGG without and with progression samples from cohort I (*p-value=0.007). (d) Log2 expression levels of miR-888-5p in PA without and with progression samples from cohort II (*p-value=0.007). (e) Normalized relative Ct expression levels of miR-1248 in pLGG without and with progression samples from cohort I (*p-value= 0.029). (f) Log2 expression levels of miR-1248 in PA without and with progression samples from cohort II (*p-value=0.0119). Figure S4. ROC curve of miR-376a-3p in pLGG with and without progression (AUC=0.5891; p-value=0.3986). Blue line=sensitivity, red line=identity. Figure S5. MiR-1248 levels distinguish Hemispheric PA and non-PA tumours with progression from Midline ones. (a) ddPCR copies/μL of miR-1248 in all the pLGG subgroups with progression. * p<0.05 vs PA Hemispheric with progression, ° p<0.05 vs GG Hemispheric with progression (b) ddPCR copies/μL of miR-1248 in Hemispheric PA with progression vs Midline PA with progression. ** p<0.01 vs PA Hemispheric with progression (c) ddPCR copies/μL of miR-1248 in Hemispheric non-PA with progression vs Midline non-PA with progression. The blue dots refer to hemispheric GG with progression, the fuchsia dot refers to the hemispheric DNET with progression, the turquoise dots refer to the midline GG with progression. * p<0.05 vs non-PA Hemispheric with progression. [file 40364_2022_389_MOESM1_ESM.pptx]

## Slide 1
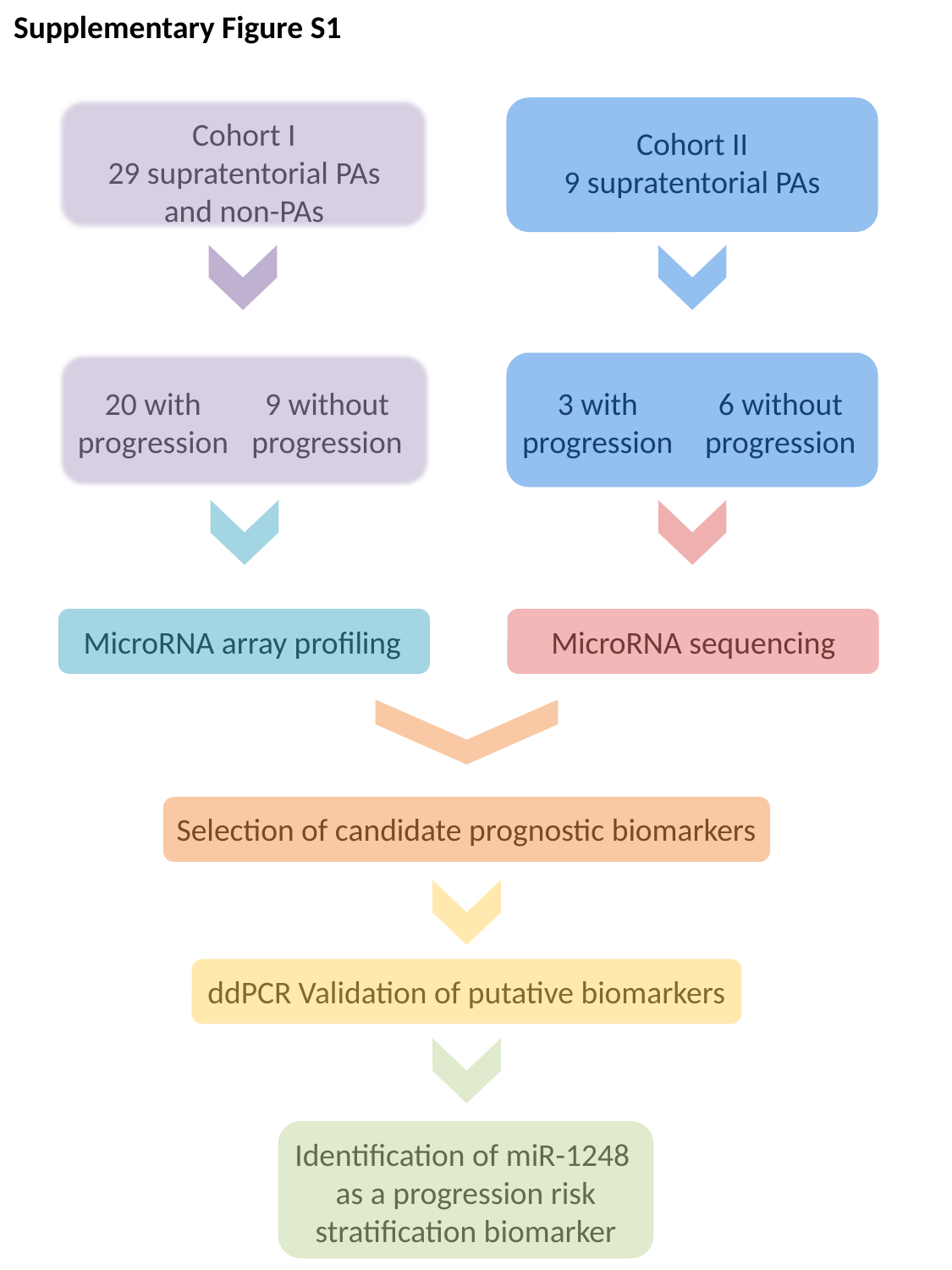

Supplementary Figure S1
Cohort I
29 supratentorial PAs and non-PAs
Cohort II
9 supratentorial PAs
20 with progression
9 without progression
3 with progression
6 without progression
MicroRNA array profiling
MicroRNA sequencing
Selection of candidate prognostic biomarkers
ddPCR Validation of putative biomarkers
Identification of miR-1248
as a progression risk stratification biomarker

## Slide 2
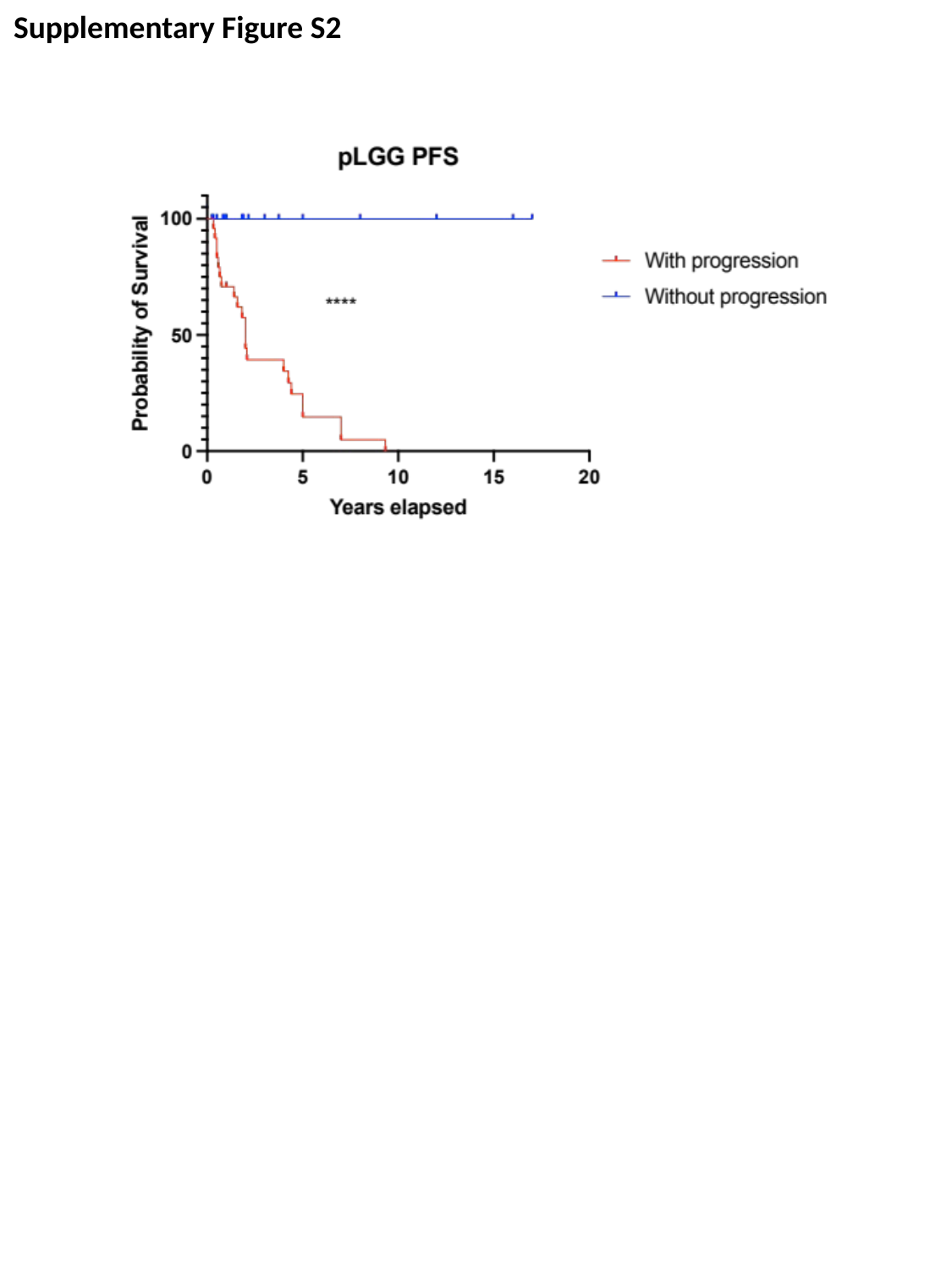

Supplementary Figure S2

## Slide 3
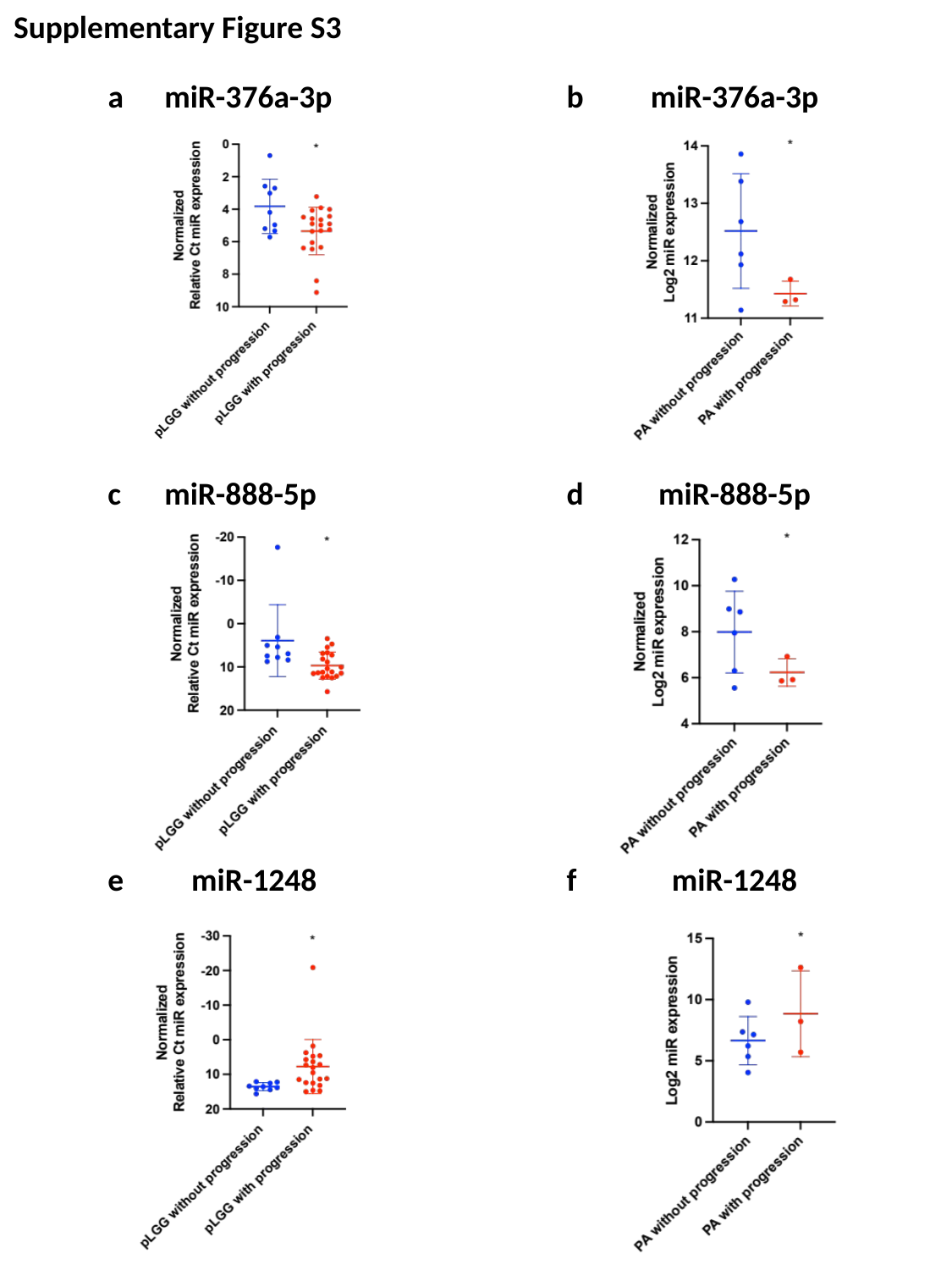

Supplementary Figure S3
a
miR-376a-3p
b
miR-376a-3p
c
miR-888-5p
d
miR-888-5p
e
miR-1248
f
miR-1248

## Slide 4
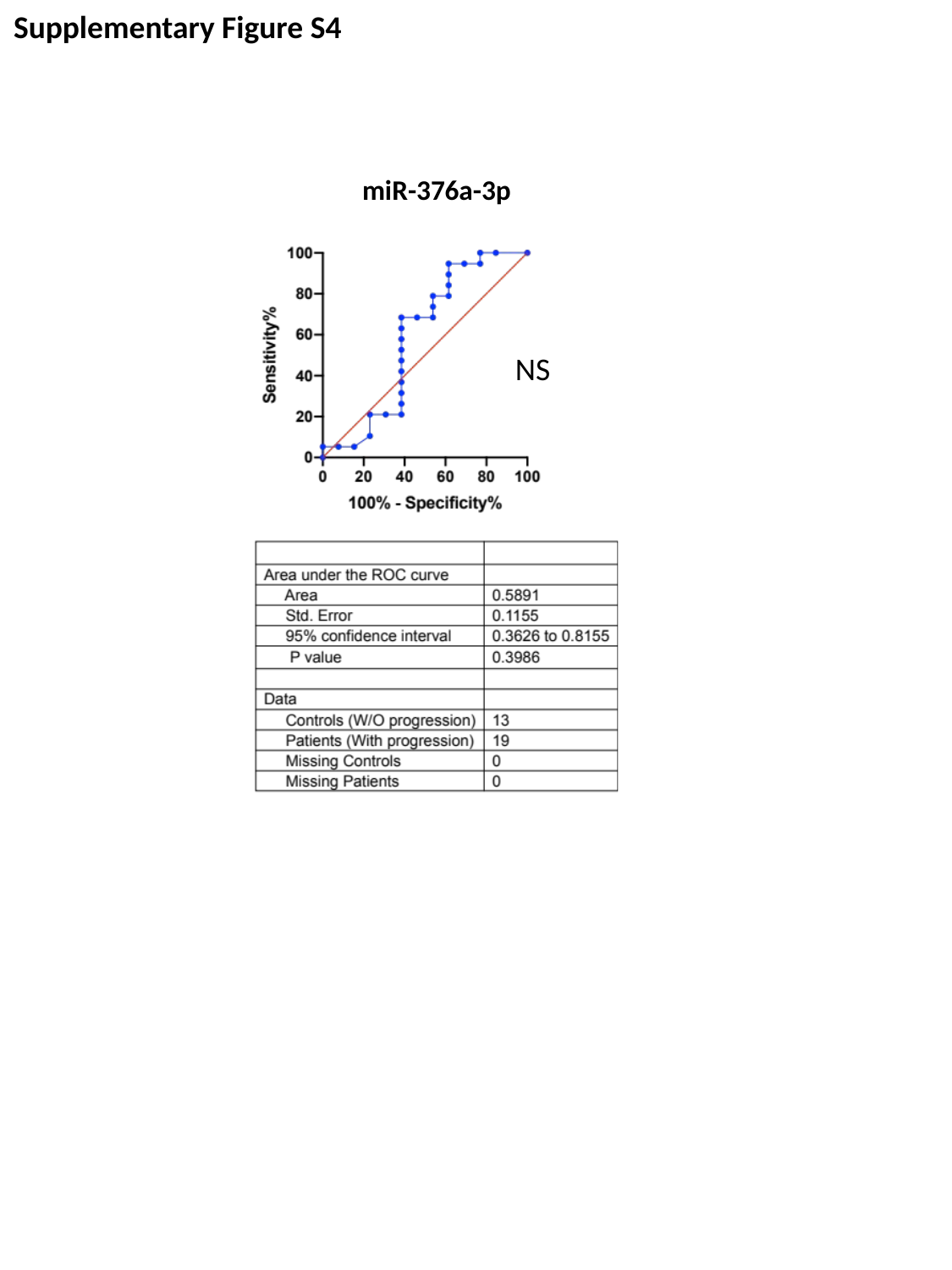

Supplementary Figure S4
miR-376a-3p
NS

## Slide 5
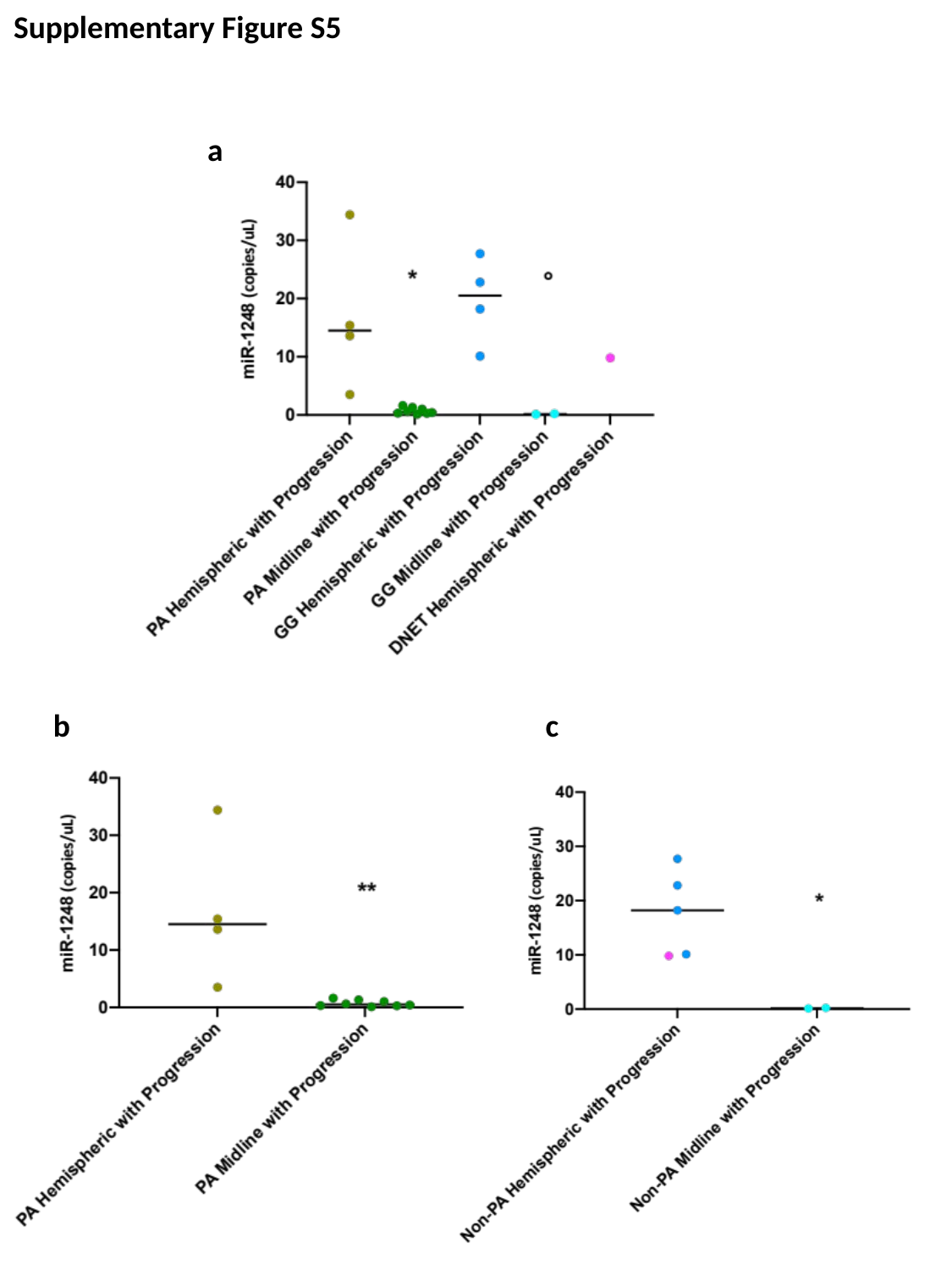

Supplementary Figure S5
a
b
c
